# Antileishmanial Activity of Bioactive Compounds Obtained from *Azadirachta indica* and *Cnidoscolus* spp.: Systematic Review

**DOI:** 10.3390/molecules31152630

**Published:** 2026-07-28

**Authors:** Jeison Alfonso Yaima-Yate, Catalina Lapuente-Chala, Daniel Alfonso Urrea-Montes, Ángel Enrique Céspedes-Rubio

**Affiliations:** 1 IMPRONTA Research Group, Faculty of Veterinary Medicine and Animal Science, Universidad Cooperativa de Colombia, Ibague 730006, Colombia; yeisson.yaima@campusucc.edu.co (J.A.Y.-Y.); clapuentec@ut.edu.co (C.L.-C.); 2 Neurodegenerative Diseases Research Group (END), Department of Animal Health, Universidad del Tolima, Ibague 730006, Colombia; 3 Tropical Parasitology Research Laboratory (LIPT), Faculty of Sciences, Department of Biology, Universidad del Tolima, Ibague 730006, Colombia; daurrea@ut.edu.co

**Keywords:** antiparasitic, biofunctional, Leishmania, phytopharmaceuticals, zoonosis

## Abstract

Leishmaniasis, a poorly studied tropical disease, has major therapeutic limitations, driving the search for safer, effective treatments. This review assessed scientific evidence on the antileishmanial activity of extracts, fractions, and bioactive compounds from *Azadirachta indica* and *Cnidoscolus* spp. in in vitro and in vivo studies. Following PRISMA 2020 guidelines, searches were conducted in PubMed/MEDLINE, Scopus, ScienceDirect, and Google Scholar. Eligible studies reported quantitative outcomes, including IC_50_/EC_50_ values, percentage inhibition, parasite load reduction, cytotoxicity, or selectivity index. Systematic reviews, studies without quantitative data, and unvalidated in silico studies were excluded. Overall, 87 records were identified, and 14 studies were included in the synthesis. Most evidence focused on *A. indica* derivatives tested against different Leishmania species, mainly in vitro, although some studies used BALB/c and CD-1, mouse models. Active fractions generally showed greater potency against intracellular amastigotes than promastigotes, with activity varying by fraction and model. In vivo studies with *A. indica* preparations reported reduced parasite loads and improved lesions. Evidence for *Cnidoscolus* spp. was limited and variable. Overall, plant-derived compounds show promising antileishmanial potential, but rigorous comparative studies are needed to strengthen evidence-based phytopharmaceutical development for leishmaniasis and to clarify the most active compounds, doses, formulations, and therapeutic targets across models.

## 1. Introduction

Leishmaniasis—a tropical disease—poses a serious public health challenge globally, regionally, and nationally. Despite its wide geographical distribution, specifically in tropical and subtropical areas, effective and safe treatments remain limited [1,2]. Cutaneous leishmaniasis (CL) is the most common clinical form of leishmaniasis. It causes skin lesions and aesthetic sequelae, resulting in social stigmatization that substantially affects the patient’s quality of life [3,4,5,6]. The rise in CL cases in Colombia over recent decades, along with persistent endemic foci and at-risk human and animal populations, emphasizes the urgent need for research and development of new therapeutic strategies for leishmaniasis [7,8,9].

Treatments available at present for leishmaniasis, mainly pentavalent antimonial compounds and second-line drugs, have some limitations, including high toxicity, emerging parasitic resistance, prolonged and costly regimens, and poor treatment adherence. [10,11]. These challenges are exacerbated in endemic regions with limited access to health services, undermining disease control efforts and further emphasizing the need for newer, safer, more effective, and more accessible therapeutic alternatives [12,13,14,15,16].

Plant-derived bioactive compounds have attracted increasing interest as potential therapeutic alternatives for leishmaniasis [17,18]. Their chemical diversity and known antiprotozoal activity against different Leishmania species in experimental studies make them a promising option for exploring new antileishmanial treatment agents. *Azadirachta indica* and *Cnidoscolus* spp., widely used in traditional medicine, are among the plants often evaluated owing to their antiparasitic, anti-inflammatory, and immunomodulatory properties at the experimental level and demonstrated in various biological models. Although several studies have evaluated the antileishmanial activity of extracts, fractions, and compounds derived from these plants, the available evidence is fragmented. Furthermore, substantial heterogeneity in experimental models, numerous Leishmania strains and parasite forms, and reported outcomes have complicated comprehensive comparative analysis [19,20,21,22,23].

To date, no systematic review has synthesized and critically evaluated the antileishmanial activity of *A. indica* and *Cnidoscolus* spp. This research gap underscores the need for a systematic literature review to comprehensively analyze the available evidence, key methodological characteristics, reported outcomes, and knowledge gaps. The present review will help guide future experimental investigations and the rational development of new therapeutic alternatives for CL, a disease with major effects on human and animal populations in endemic regions, specifically in Colombia [24,25,26,27,28,29].

## 2. Results

**Figure 1:**
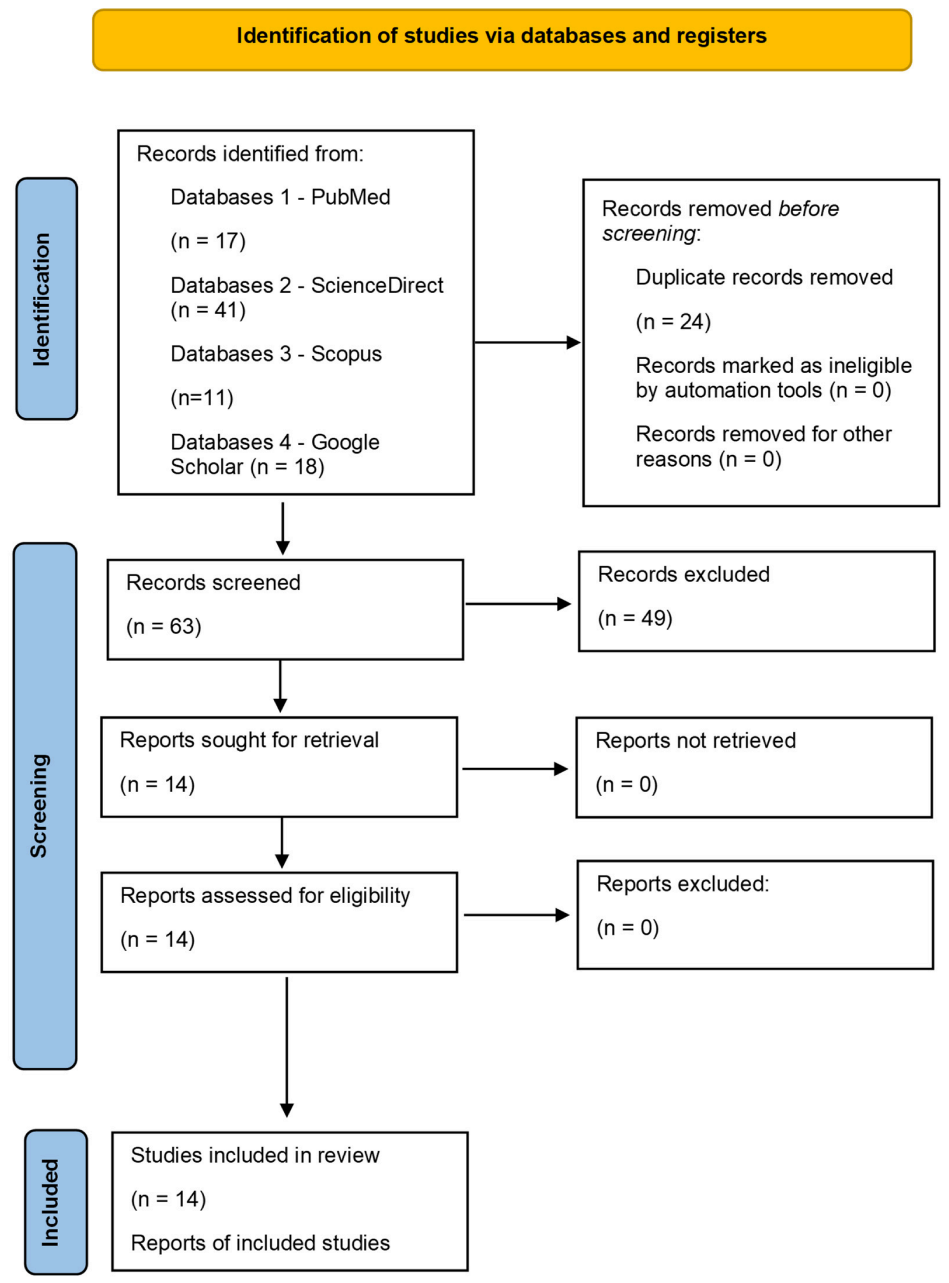
Flowchart of the identification and selection of antileishmanial activity studies on *A. indica* and *Cnidoscolus* spp. Source: Page MJ et al. BMJ 2021;372: n71. https://doi.org/10.1136/bmj.n71 [30].

The study identification and selection process are summarized in the PRISMA diagram (Figure 1). Overall, 85 records were retrieved from the electronic database searches. After eliminating 22 duplicates, 63 unique records published between 2005 and 2025 were screened by title and abstract. In this phase, 49 were excluded for not meeting the eligibility criteria, and 14 articles were selected for full-text evaluation.

No studies were excluded after full-text evaluation because the 14 publications included for full-text review met the inclusion criteria.

The 14 experimental studies included were conducted in India, Mexico, Brazil, Cuba, Sudan, Kenya, Italy, Suriname, and the Netherlands, reflecting a broad geographic distribution of the available evidence.

Regarding experimental design, seven studies used only in vitro models, whereas five combined in vitro and in vivo models. One study was performed exclusively in vivo, whereas only one included an in silico analysis. The in vitro models evaluated antileishmanial activity mainly against promastigotes and amastigotes, including intracellular amastigotes in cell lines such as THP-1, RAW 264.7, J774.2, and Vero. In vivo studies used BALB/c or CD-1 mice.

*Leishmania* spp. evaluated included *Leishmania donovani*, *Leishmania major*, *Leishmania mexicana*, *Leishmania amazonensis*, *Leishmania infantum*, *Leishmania tropica*, *Leishmania braziliensis*, and *Leishmania guyanensis*. The interventions analyzed included crude extracts, organic fractions, commercial products, and different fractions derived mainly from *A. indica* (leaves, seeds, fruits, and neem oil) and *Cnidoscolus* spp. (leaves).

Outcomes included antileishmanial activity, cytotoxicity, and SI in nine studies, and immunomodulatory and/or anti-inflammatory effects in five studies. The individual characteristics of each study are presented in Table 1.

Overall, the 14 studies included showed a predominantly moderate overall risk of bias, with multiple domains classified as uncertain due to incomplete methodological reporting. The main limitation of the in vitro studies evaluated using the QUIN Tool was related to the absence of explicit information on evaluator blinding and, in some cases, a lack of details on experimental replication. In the in vivo studies evaluated using SYRCLE, several domains were categorized as uncertain due to a lack of randomization reporting, allocation concealment, and blinding during outcome assessment. Nevertheless, most of the papers reported appropriate pharmacological controls, which partially strengthens the internal validity of the findings.

### 2.1. A. indica (Neem)

*A. indica* was the most extensively studied plant source, with numerous in vitro evaluations against promastigotes and/or amastigotes (including intracellular forms) as well as several in vivo studies in murine models, mainly involving *L. donovani* (visceral leishmaniasis) and *L. major* (cutaneous).

- In vitro activity (promastigotes and amastigotes)

Vijayakumar et al. (2025) [31] reported azadiradione (AZD) activity against *L. donovani* with IC_50_ = 17.09 μM against promastigotes and EC_50_ = 11.67 μM against intracellular amastigotes. Furthermore, they reported CC_50_ = 56.32 μM in THP-1 and SI = 4.83, indicating moderate selectivity. Cesa et al. (2019) [34] reported low activity of *A. indica* seed oil against promastigotes (*L. infantum* IC_50_ = 215 μg/mL; *L. tropica* IC_50_ = 211 μg/mL) but greater activity against intracellular amastigotes, with IC_50_ = 15.3 μg/mL (*L. infantum*) and 17.6 μg/mL (*L. tropica*). Furthermore, they reported a CC_50_ = 710.5 μg/mL and SI = 46 and 40, respectively. Mans et al. (2016) [35] found limited and variable activity for the aqueous leaf extract, depending on the Leishmania species. Against promastigotes, the extract showed IC_50_ > 4500 μg/mL for *L. guyanensis*, IC_50_ > 187 μg/mL for *L. major* and IC_50_ > 247 μg/mL for *L. donovani*. Against intracellular *L. donovani* amastigotes (BHU-814), IC_50_ = 451 μg/mL and CC_50_ = 352 μg/mL were observed, indicating low potency and unfavorable selectivity under the study’s experimental conditions.

Dayakar et al. (2015) [36] reported IC_50_ = 52.4 μg/mL for the ethyl acetate fraction against *L. donovani* promastigotes. Conversely, for intracellular amastigotes, they found an approximately 45–50% reduction in parasite load with an IC_50_ ≈ 10 μg/mL. Chouhan et al. (2015) [21] evaluated the activity of leaf and seed fractions against *L. donovani* (AG83). In promastigotes, the leaf fraction showed an IC_50_ of 34 μg/mL, compared with 77.66 μg/mL for the seed fraction. Against intracellular amastigotes, the leaf fraction showed IC_50_ = 17.66 μg/mL, SI = 26.10 and CC_50_ = 461 μg/mL, whereas the seed fraction had an IC_50_ of 24.66 μg/mL and CC_50_ >500 μg/mL, being reported as non-toxic.

García et al. (2012) [38] studied the hydroalcoholic extract of *A. indica* leaves, finding no relevant activity against *L. amazonensis* promastigotes (IC_50_ > 200 μg/mL). Consequently, evaluation against amastigotes was not conducted, based on their internal selection criteria (SI > 5). Carneiro et al. (2012) [20] found a clear gradient of potency, with the ethanolic leaf extract achieving IC_50_ = 38.0 μg/mL against promastigotes and IC_50_ = 9.8 μg/mL against intracellular amastigotes (with CC_50_ = 56.96 μg/mL and SI = 5.81). The lipophilic fractions yielded the best results, with the CH_2_Cl_2_-l fraction achieving IC_50_ = 1.1 μg/mL against amastigotes with (CC_50_ = 48.1 μg/mL and SI = 43.72), and the CHCl_3_-c fraction achieving IC_50_ = 0.6 μg/mL against amastigotes with high selectivity (SI > 20).

Singh et al. (2011) [39] reported complete elimination of *L. donovani* promastigotes at a minimum effective concentration (MEC) = 0.1 mg/mL within 48 h. Furthermore, axenic amastigotes were 1.9 times more susceptible than promastigotes. Similarly, Khalid et al. (2005) reported an LC_50_ = 10.21 μg/mL against *L. major* promastigotes but provided no cytotoxicity/selectivity parameters [40].

b. In vivo activity

Dayakar et al. (2015) [36] reported a reduction in visceral parasite load (Leishman–Donovan Units [LDU]) in a BALB/c model infected with *L. donovani*. Following treatment, LDU decreased from 243.5 to 98.7 in the spleen and from 4923.8 to 1354.6 in the liver.

Chouhan et al. (2015) [21] reported high protection rates against parasite load (LDU), with high values for leaf and seed fractions (leaves: ~87.76% for liver and 85.55% for spleen; seeds: ~85.54% for liver and 83.62% for spleen).

Jumba et al. (2015) [37] evaluated methanolic extracts of *A. indica*, both alone and in combination with *Ricinus communis*. The *A. indica* extract alone achieved an IC_50_ = 10.1 μg/mL against promastigotes and IC_50_ = 11.5 μg/mL against amastigotes, with a CC_50_ of 149 μg/mL in Vero cells. Notably, the combined treatment with *R. communis* improved efficacy, yielding an IC_50_ = 9.0 μg/mL against amastigotes. Kaur et al. (2013) [2] observed a substantial reduction in liver parasite load (LDU) with *A. indica*, with a greater effect when combined with *Emblica officinalis*. However, the IC_50_ values were not reported.

Overall, the in vivo and in vitro studies demonstrate significant potential antileishmanial properties of *A. indica* against different *Leishmania* spp. The in vitro activity demonstrated by *A. indica* showed a consistent trend toward greater efficacy against intracellular amastigotes than against promastigotes. Several studies reported low IC_50_ values and high SIs, specifically those investigating lipophilic fractions or isolated compounds. In vivo studies using BALB/c murine models demonstrated significant reductions in visceral and hepatic parasite load, as well as immunomodulatory effects, reinforcing the potential therapeutic relevance of *A. indica* against Leishmania infections.

### 2.2. Cnidoscolus *spp.* (Pringamoza)

Reports on *Cnidoscolus* spp. are rare and mostly focus on in vitro studies against *L. mexicana* and *L. braziliensis*, evaluating crude extracts and isolated compounds, with heterogeneous results.

López-Huerta et al. (2020) [22] Isolated hopanes 2 and 3 (3-oxo-hop-22(29)-ene and 3β-hydroxy-hop-22(29)-ene, respectively), from CH_2_Cl_2_: MeOH extract from *C. spinosus*, exhibited antileishmanial activity against *L. mexicana* promastigotes. However, IC_50_ values were not explicitly reported. Juárez-Vázquez et al. (2020) [32] reported that the hexane extract of *Cnidoscolus tehuacanensis* was more active than the isolated biocompound lupeol acetate. The extract had an IC_50_ = 58.98 μg/mL (SI = 2.11) against *L. mexicana* promastigotes and IC_50_ = 8.73 μg/mL (SI = 14.31) against amastigotes. Conversely, lupeol acetate showed lower potency (promastigotes: IC_50_ = 82.02 μg/mL; amastigotes: IC_50_ = 52.33 μg/mL). Fernandes et al. (2020) [33] reported that linamarin extracted from *Cnidoscolus quercifolius* did not inhibit *L. braziliensis* promastigotes within a concentration range of 31.25–2000 μg/mL. Thus, the IC_50_ could not be calculated. A summary of the reports is presented in Table 2.

The studies that evaluated the antileishmanial activity of *A. indica* and *Cnidoscolus* spp. reported heterogeneous results. This variability was mainly associated with methodological and biological differences between experimental designs, including the type of plant preparation used, crude extracts, fractions, and isolated compounds. Generally, extracts showed higher variability in activity values (IC_50_/EC_50_), possibly due to phytochemical complexity and potential synergistic effects between secondary metabolites.

Heterogeneity was influenced by the Leishmania species and parasite stage evaluated. The different Leishmania species investigated included *L. guyanensis*, *L. amazonensis*, *L. braziliensis*, *L. major*, *L. mexicana*, *L. donovani*, *L. tropica*, and *L. infantum*, all of which have intrinsic differences in pharmacological susceptibility. Furthermore, different cellular and animal models were used. In vitro assays were conducted using murine (RAW 264.7), human (THP-1), and primary macrophage cell lines, whereas in vivo models mainly used BALB/c mice. These variations can modify the immune response, phagocytic capacity, and pharmacodynamics of the compounds evaluated.

Another important source of heterogeneity was the diversity in reported outcome measures, which included IC_50_, EC_50_, LC_50_, MEC, and LDU.

To clearly map the complex multi-dimensional data underlying plant-derived antileishmanial research, we designed a circular visualization scheme (The Circos-based visualization) using the circlize package in the R environment. Traditional systematic review tables are useful, but they tend to fragment interrelated variables. This fragmentation makes it difficult to understand the effects of specific extraction methods on parasite inhibition at different life stages. Our circular layout closes these gaps by following a clear evidence trail from the raw botanical source down to its final biological potency (IC_50_). Such holistic mapping, combining plant parts, extract fractions, Leishmania species, and developmental stages in a unified design, reveals taxonomic hotspots, neglected chemical fractions, and high-efficacy clusters.

**Figure 2:**
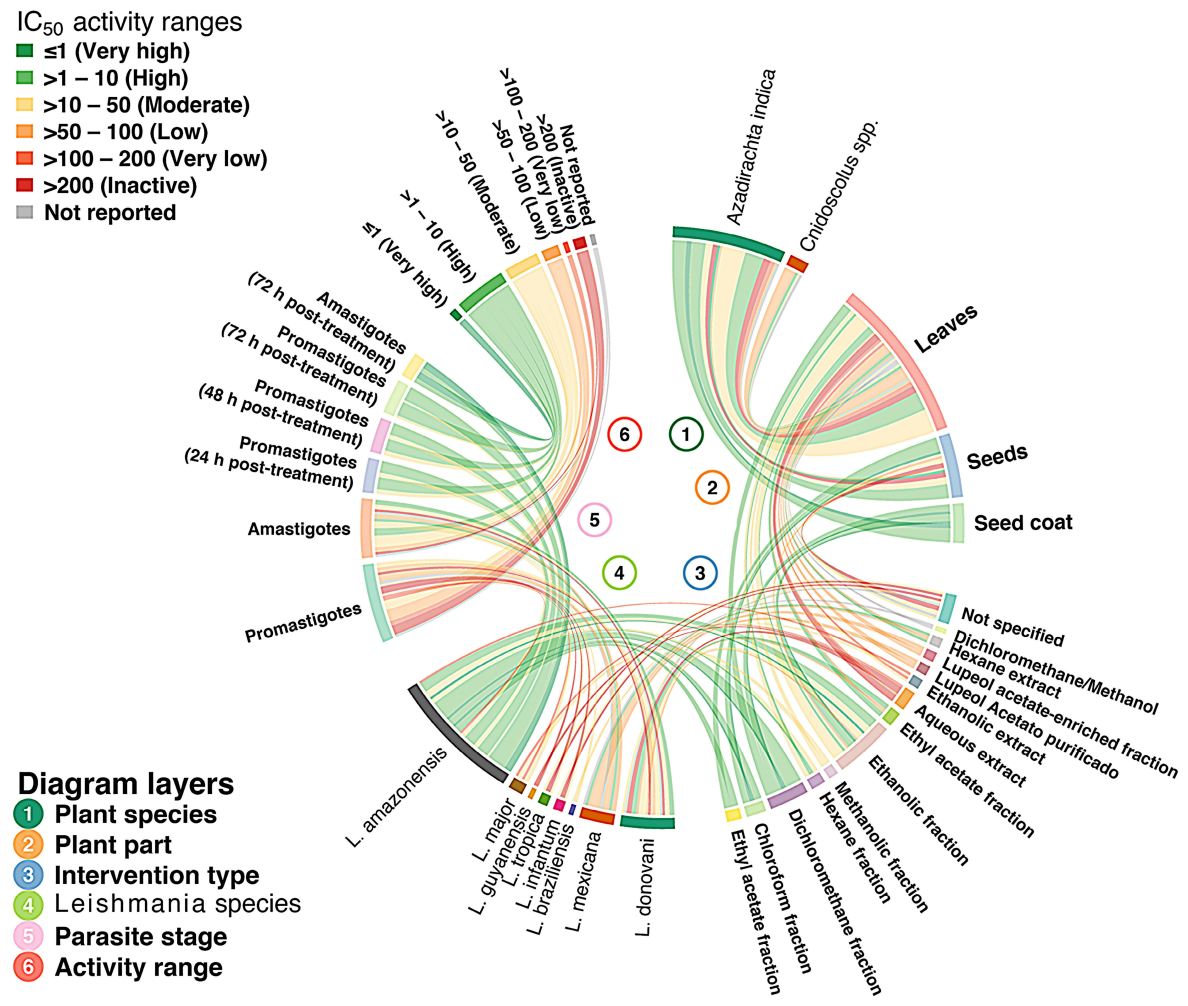
The Chord Diagram shows how plant species (*Azadirachta indica* and *Cnidoscolus* spp.) and their respective plant parts (leaves, seeds, and seed coat) are connected to the different types of interventions used against Leishmania parasites (crude extracts and fractions derived from solvents). This diagram depicts the various species of Leishmania (i.e., *L. amazonensis*, *L. guyanensis*, *L. major*, *L. infantum*, *L. tropica*, *L. braziliensis*, *L. mexicana*, and *L. donovani*) at different stages of the Leishmania life cycle (promastigotes and amastigotes) using different methods of measurement based on time post-treatment and a statistically significant (IC_50_) value. The ranges of antileishmanial activity have been classified called “very high” (≤1 µg/mL); “high” (>1–10 µg/mL); “moderate” (>10–50 µg/mL); “low” (>50–100 µg/mL); “very low” (>100–200 µg/mL); “inactive” (>200 µg/mL); and “unreported”. The width of each string connecting plant species to their respective type of intervention will be dependent on how many times such associations were recorded from the literature that was reviewed. There are six concentric layers in the chord diagram: (1) plant species; (2) part of the plant where the extract was derived; (3) type of extract used; (4) species of Leishmania; (5) stage of the parasite; and (6) range of antileishmanial activity based on the IC_50_ value.

The predominance of *A. indica-derived* data was observed, with multiple connections spanning different plant parts (mainly leaves and seeds), extraction types, and Leishmania species, indicating a broader experimental exploration compared to *Cnidoscolus* spp. Notably, the strongest connections were associated with amastigote stages, particularly intracellular amastigotes, which were more frequently linked to higher activity ranges (IC_50_ ≤ 10 μg/mL and 1–10 μg/mL). In contrast, promastigote-related interactions were more widely distributed across moderate to low activity ranges. Lipophilic extracts and organic fractions, especially those derived from leaves, showed a higher frequency of associations with potent activity levels, whereas aqueous extracts and non-specified preparations were more commonly linked to lower or variable activity. Additionally, the visualization highlights a heterogeneous distribution of activity across Leishmania species, with *L. donovani* and *L. amazonensis* showing the highest density of experimental links (Figure 2).

Table 3 summarizes the in vitro antileishmanial activity of leaf-derived extracts and fractions from *Cnidoscolus* spp. against different Leishmania species, mainly *L. mexicana* and *L. braziliensis*. The biological activity varied according to the type of extract, fraction, and parasite developmental stage evaluated. Dichloromethane/methanol extracts exhibited activity against promastigotes of *L. mexicana*, although no IC_50_ values were reported.

Among the evaluated fractions, the hexane extract showed the highest activity against amastigote forms of *L. mexicana* in murine macrophages J774.2, with IC_50_ values ranging between 1 and 10 µg/mL, indicating high antileishmanial activity. In contrast, the same extract displayed lower activity against promastigotes (>50–100 µg/mL). Similarly, lupeol acetate-rich fractions and purified lupeol acetate demonstrated low activity against both promastigote and amastigote stages, suggesting that the isolated compound may not fully account for the stronger activity observed in the crude hexane extract. Ethanolic extracts exhibited moderate activity against promastigotes of *L. braziliensis*, with IC_50_ values between 10 and 50 µg/mL. Overall, these findings suggest that non-polar extracts from *Cnidoscolus* spp. leaves may contain bioactive metabolites with promising antileishmanial potential, particularly against intracellular amastigote forms, which represent the clinically relevant stage of infection.

Table 4 summarizes the antileishmanial activity of different extracts and fractions derived from *Azadirachta indica* seed oil against several Leishmania species, including *L. infantum*, *L. tropica*, *L. donovani*, and *L. amazonensis*. The results demonstrate that the biological activity varies considerably depending on the extract polarity, parasite species, and developmental stage evaluated.

Commercial seed oil exhibited moderate activity against intracellular amastigote forms of *L. infantum* and *L. tropica* in THP-1 human monocytic cells, with IC_50_ values ranging between 10 and 50 µg/mL. However, the same oil was practically inactive against promastigote forms of both species, showing IC_50_ values greater than 200 µg/mL. These findings suggest that the bioactive compounds present in the seed oil may exert stronger effects on intracellular parasite stages than on extracellular promastigotes.

The ethanolic fraction showed low activity against promastigotes of *L. donovani*, whereas moderate activity was observed against amastigotes in RAW 264.7 murine macrophages. This difference again highlights the higher susceptibility of intracellular amastigote forms to certain phytochemical constituents of *A. indica*.

Among all evaluated samples, the chloroform fraction demonstrated the most promising antileishmanial effect. This fraction exhibited high activity against promastigotes of *L. amazonensis* at 24, 48, and 72 h post-treatment, with IC_50_ values between 1 and 10 µg/mL. Moreover, very high activity (≤1 µg/mL) was observed against intracellular amastigotes after 72 h, indicating potent leishmanicidal activity. Similarly, the ethyl acetate fraction also showed high activity against promastigotes of *L. amazonensis* throughout the evaluated time points.

Overall, the results indicate that non-polar and semi-polar fractions from *A. indica* seed oil, particularly chloroform and ethyl acetate fractions, possess significant antileishmanial potential. The stronger activity observed against amastigote stages is especially relevant because this intracellular form is responsible for disease progression in mammalian hosts. These findings support the potential use of *A. indica*-derived bioactive compounds as promising candidates for the development of alternative antileishmanial therapies.

## 3. Discussion

**Figure 3:**
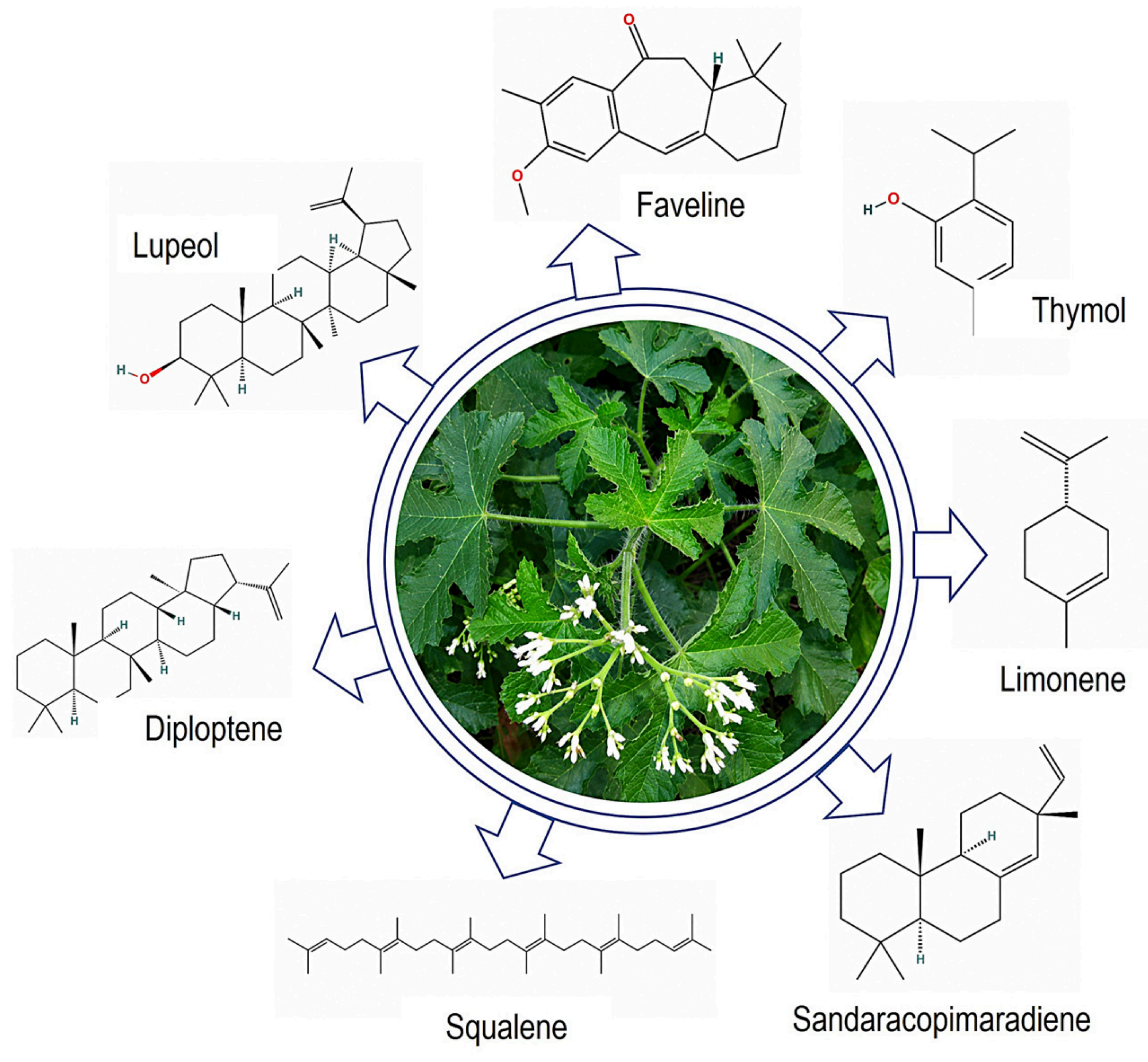
Representative phytochemical compounds identified in *Cnidoscolus* spp. The figure highlights the structural diversity of major compounds described in *Cnidoscolus* species and their potential as sources of novel antileishmanial agents.

The in silico analyses by Barrera-Tellez et al. (2023) [41] and Samson et al. (2026) [42] indicate that *A. indica* and, to a lesser extent, *Cnidoscolus* spp. contain bioactive compounds with potential antileishmanial activity (Figure 3). These compounds interact specifically with the trypanothione reductase enzyme, inhibiting its function, thus affecting survival at the intracellular amastigote stage, which constitutes the clinically relevant form of the parasite in infected patients [43,44]. These results highlight the increasing interest in the biological activity of various natural products, plant extracts, and secondary metabolites as supplementary or complementary therapeutic alternatives for the treatment of leishmaniasis, especially considering the current therapeutic limitations [45].

Compounds from the hexane fraction may exhibit a higher potency than isolated metabolites, whereas other isolated compounds, such as linamarin, may exhibit no activity under the conditions under study. This review confirms that the evidence remains limited, with outcomes reported unevenly across studies. Overall, the 14 studies included suggest considerable heterogeneity in interventions (extracts, fractions, and isolated compounds), Leishmania species/strains, experimental models, and outcomes. However, evidence on the activity of *A. indica* is more extensive. Several studies have reported significant activity against intracellular amastigotes and signs of in vivo efficacy (LDU reduction), especially for enriched fractions [46]. Conversely, *Cnidoscolus* spp. have fewer publications with variable results and findings that strongly depend on the type of extract/compound evaluated [47,48,49].

Regarding available therapies, Fan et al. (2025) [50] and Frézard et al. (2023) [51] noted that conventional treatments with pentavalent antimonials, amphotericin B, or miltefosine are associated with significant toxicity, high costs, the need for parenteral administration, and the emergence of resistant target strains [52]. However, Singh et al. (2022) [53], Tandon et al. (2021) [54], and Koko et al. (2022) [55] reported that medicinal plant products offer promising alternatives and may contain novel active compounds with more favorable safety profiles [56]. The evidence synthesized in this review partially supports this perspective, demonstrating that some fractions, mainly limonoids and triterpenoids isolated from *A. indica*, have comparable inhibition values in terms of magnitude to reference drugs in experimental models. This statement is further supported by Vijayakumar et al. (2025) [31] and Ali et al. (2021) [44], who emphasize the importance of natural therapies. In line with this, our study found that the lipophilic fractions of leaves and seeds exhibited the highest antileishmanial potency. This has been described as a fundamental strategy in the early stages of natural product-based drug discovery [57,58,59,60].

Other previous reports describe the antiparasitic activity of plant extracts, being greater on amastigotes than on Leishmania promastigotes, probably due to metabolic differences and interaction with host cells of these forms and parasite stages [61,62,63,64]. This variability aligns with the following known factors: extraction, plant organ, phytochemistry, and Leishmania species [65,66]. Furthermore, the evidence reviewed indicates that many natural compounds with antileishmanial activity exert their effects on the parasite through apoptotic and oxidative stress mechanisms. Balamurugan et al. (2022) [24] noted that flavonoids and other metabolites induce apoptosis, DNA damage (genotoxicity), and altered redox metabolism in *L. donovani* [67,68,69].

Thus, experimental findings suggesting that neem fractions induce parasitic apoptosis are biologically plausible and consistent with mechanisms reported for other bioactive natural compounds [60,70,71,72]. This is because various secondary metabolites—not only of *A. indica* but of plant derivatives in general—such as flavonoids, alkaloids, terpenoids, and polyphenols induce apoptosis-like death in *Leishmania* spp. Specifically, these compounds induce the overproduction of reactive oxygen species, loss of mitochondrial membrane potential, phosphatidylserine exposure, DNA fragmentation, and cell cycle alterations [47,48,49,60,72].

**Figure 4:**
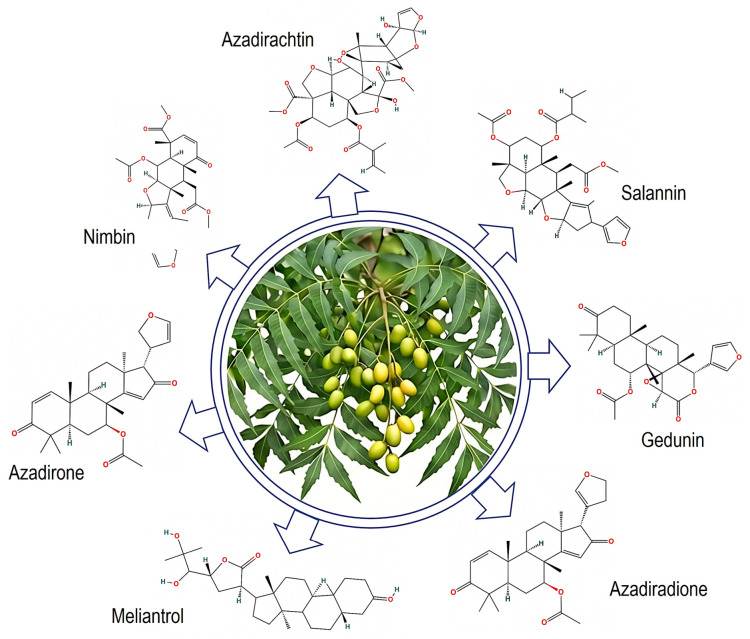
Representative phytochemical compounds isolated from *Azadirachta indica* (Neem). These metabolites mainly belong to the limonoid and tetranortriterpenoid groups.

*A. indica* contains a wide variety of limonoids, including azadirachtin and nimbin, as well as other phenolic compounds and terpenoids (Figure 4) with recognized redox potential and ability to interact with parasitic organelles. Thus, their active fractions may trigger programmed cell death pathways through mechanisms associated with oxidative stress and mitochondrial dysfunction [73]. Therefore, the induction of antileishmanial apoptosis not only reinforces the coherence of the findings of the studies evaluated in this review, but it also categorizes *A. indica* fractions within a widely documented mechanistic framework of antileishmanial natural products. This evidence strengthens their pharmacological and biomedical relevance as bioactive candidates [21,36,47].

In contrast to the scientific publications on *A. indica*, the evidence reviewed on *Cnidoscolus* spp. is more limited and rather heterogeneous. Although some studies, such as that by López-Huerta et al. (2020) [22], reported that Cnidoscolus extracts exhibit relevant activity against *L. mexicana*, others, such as that by Juárez-Vázquez et al. (2020) [32], demonstrate no such antiparasitic inhibition. This evidences that the therapeutic potential of Cnidoscolus derivatives should be further explored so that robust conclusions can be drawn [33]. Posada-Mayorga et al. (2024) [43] reported that an integrative analysis of Cnidoscolus shows that, despite several species containing bioactive triterpenes and sterols, their pharmacological validation against protozoa remains limited. This supports the scarce and heterogeneous results observed in this review, where some fractions proved more potent than isolated metabolites, such as lupeol acetate. These findings suggest that synergistic interactions within phytochemical mixtures play a decisive role, a concept supported by recent literature [57,74,75].

Collectively, our review provides comprehensive evidence positioning *A. indica* as a leading candidate in preclinical antileishmanial development, specifically against intracellular amastigotes and in murine models of visceral leishmaniasis. However, consistent with international reviews, most of these findings correspond to early experimental stages. Consequently, translating these results into therapeutic applications will require methodological standardization, rigorous chemical characterization, and future clinical trials [76,77,78,79,80,81].

Although the included studies offer valuable preliminary evidence on the antileishmanial activity of *A. indica* and some *Cnidoscolus* spp., they are based exclusively on in vitro and in vivo studies. No human clinical trials have yet been conducted [82]. Therefore, direct therapeutic applicability of the results is limited, as the experimental models do not fully reproduce the immunological, pharmacokinetic, and pathophysiological complexity of infection under clinical conditions [83,84,85,86]. Furthermore, considerable methodological heterogeneity was observed across studies. The studies differed in the Leishmania species and strains, number of parasite stages (promastigotes, axenic, and intracellular amastigotes), host cell lines, and animal models investigated. Furthermore, the products evaluated varied widely across crude extracts, organic fractions, commercial products, and isolated compounds, with non-standardized extraction methods [87,88,89,90,91].

Another major contributing factor to the heterogeneity was the inconsistent reporting of outcomes. Although several studies reported IC_50_ or EC_50_ values, others measured only MEC, LC_50_, or % inhibition. Precision measures or confidence intervals were not reported. Furthermore, not all the studies conducted comprehensive evaluations of cytotoxicity or selectivity indices, which restricted efficacy-safety balance interpretations [18,90,92].

Furthermore, the risk of bias was moderate to high in several studies, mainly due to a lack of information on randomization, experimental blinding, sample size calculation, and comprehensive reporting of results. Although common in early experimental investigations, these methodological limitations reduce the overall robustness of the body of evidence. Moreover, since studies reporting negative effects or lack of activity may be underrepresented in the literature, the possibility of publication bias and missing results should be considered. Collectively, these limitations highlight the need for further, more rigorous investigations that strictly implement standardization, thorough phytochemical characterization, robust experimental designs, and progression to controlled clinical studies [77,78,93].

The integrated analysis of the data shown in the circle diagram reinforces the key patterns observed in the narrative synthesis, particularly the central role of *A. indica* as the species with the most studies reported in this review, showing a stronger association with antileishmanial activity, especially against intracellular amastigotes. This pattern supports the biological relevance of targeting the clinically significant stage of the parasite and aligns with previous reports indicating increased susceptibility of amastigotes compared to promastigotes [25,62]. Moreover, the clustering of higher activity ranges with lipophilic fractions suggests that compound polarity plays a critical role in bioactivity, likely reflecting improved membrane permeability and interaction with intracellular targets such as trypanothione reductase [41,42]. In contrast, the dispersed and less connected profile of *Cnidoscolus* spp. highlights the limited and heterogeneous nature of current evidence for this genus, reinforcing the need for further standardized experimental studies [43].

The findings of this systematic review have substantial implications for translational research and the development of new therapeutic alternatives against visceral and mucocutaneous leishmaniasis. These insights are relevant not only for human health but also for veterinary medicine [94,95,96]. In this regard, these studies offer an important biological rationale for advancing the research from in vitro and in vivo studies to more complex preclinical models. This shift is necessary to provide technical-scientific evidence that further supports the validation of candidate compounds against resistant strains, addressing the toxicity, cost, and efficacy issues associated with conventional treatments [97,98,99].

This review advocates for better multidisciplinary collaborations, integrating experimental pharmacology, nanotechnology, and molecular mechanistic studies. This study will help bridge the gap between the descriptive characterization of bioactive principles and their application as clinically viable therapeutic candidates [98,100,101].

## 4. Materials and Methods

A systematic review of the literature was conducted to synthesize the available experimental evidence on the antileishmanial activity of extracts, fractions, and bioactive compounds derived from *A. indica*, *Cnidoscolus urens*, and other *Cnidoscolus* spp. This review was conducted in accordance with the recommendations of the Preferred Reporting Items for Systematic Reviews and Meta-Analyses (PRISMA) 2020 statement for systematic reviews [30,102].

### 4.1. Eligibility Criteria

Studies were included if they met the following criteria: (a) type of study—original experimental in vitro and/or in vivo studies; (b) object of study—evaluation of the antileishmanial activity of extracts, fractions, and/or bioactive compounds derived from *A. indica*, *C. urens*, and/or other *Cnidoscolus* spp.; (c) biological model—research conducted on *Leishmania* spp., including different strains and parasitic forms (promastigotes and/or intracellular amastigotes); (d) outcomes—reporting of at least one quantitative outcome of antileishmanial activity, such as inhibitory concentration 50/effective concentration 50 (IC_50_/EC_50_), % inhibition, reduction in parasite load or lesion size, and cytotoxicity parameters, which include cytotoxic concentration 50 (CC_50_) and selectivity index (SI); (e) language—studies published exclusively in English; and (f) availability—full-text and open access articles [103,104].

Exclusion criteria were as follows: (a) narrative or systematic reviews, meta-analyses, editorials, letters to the editor, congress abstracts, or theses; (b) studies with plants other than *A. indica* and *C. urens*, or any *Cnidoscolus* spp., or combinations of these plants without individualized results; (c) studies that did not evaluate biological activity against *Leishmania* spp.; (d) studies focused exclusively on molecular mechanisms, in silico modeling, or formulations without reporting independent biological results; (e) publications without extractable quantitative data; and (f) duplicate reports or secondary publications of the same study (only the most complete version was retained) [105].

### 4.2. Data Sources

A systematic search was conducted on PubMed/MEDLINE, ScienceDirect, Scopus, and Google Scholar [106]. Furthermore, the references listed in the included articles were retrieved and reviewed to identify other relevant publications that did not appear in the electronic searches [102,107]. The last database search for sources was conducted on 23 January 2026.

### 4.3. Search Strategy

The search strategy used in this study was a combination of botanical and other terms directly related to leishmaniasis and antileishmanial activity. For example, the following strategy was used in PubMed: (“*Azadirachta indica*” OR “neem” OR “*Cnidoscolus urens*” OR “*Cnidoscolus* spp.” OR “pringamoza”) AND (“Leishmania” OR “leishmaniasis” OR “antileishmanial”). Similar strategies were adapted for ScienceDirect, Scopus, and Google Scholar. The first 200 results retrieved from Google Scholar were ordered by relevance and subsequently reviewed [107].

### 4.4. Study Selection Process

Phase 1. Screening of titles and abstracts after eliminating duplicates.

Phase 2. Full-text evaluation of potentially eligible articles for final inclusion.

The entire study selection process was performed by a single reviewer. Record management and duplicate identification were performed using Mendeley Reference Manager version 2.145.0 (Elsevier, Amsterdam, The Netherlands) https://www.mendeley.com/ [108]. No automation tools or machine learning algorithms were used. In cases of doubt during the initial screening, the relevant studies were retained for full-text evaluation.

### 4.5. Data Extraction

Data were extracted from full texts. For each study, the following were systematically collected: (a) general characteristics of the study (author, year, country); (b) experimental model used (in vitro and/or in vivo); (c) species, strain, and parasitic form of Leishmania studied; (d) type of plant preparation evaluated (crude extract, fraction, or isolated compound); (e) quantitative outcomes of antileishmanial activity (IC_50_/EC_50_, % inhibition, parasite load reduction, lesion size); and (f) cytotoxicity parameters (CC_50_, SI), when available [109]. None of the authors of the primary studies were contacted for additional information.

### 4.6. Bias Risk Assessment

Bias risk was assessed in the studies included after full-text review using established methodological tools according to the type of experimental design [110,111].

In vivo studies in animal models were evaluated using the SYRCLE Risk of Bias tool [30,110]. In vitro studies were assessed qualitatively using methodological criteria based on the QUIN Tool [105,110,112]. Information was incompletely reported in several domains, especially those related to randomization and blinding; therefore, these domains were classified as having uncertain risk [113,114].

### 4.7. Effect Measurements and Synthesis of Results

The effect measures corresponded to the quantitative outcomes reported in the individual studies: IC_50_/EC_50_ values, % inhibition, parasite load reduction, lesion size, CC_50_ and SI, when available. Owing to the high heterogeneity among experimental designs, *Leishmania* spp., plant preparations, and units of measurement, results were synthesized using a narrative and descriptive approach [109]; no quantitative meta-analyses were performed.

### 4.8. Grouping of Studies

The studies included for analysis were grouped for synthesis to facilitate a structured and coherent presentation of the available evidence, according to: (a) type of study (in vitro and/or in vivo); (b) plant species evaluated (*A. indica* or *C. urens*, and *Cnidoscolus* spp.); (c) type of extract, fraction, or compound evaluated; and (d) species and parasitic form of Leishmania investigated [79].

Antileishmanial activity data reported in the literature for *Cnidoscolus* spp. and *Azadirachta indica* were systematically organized according to plant part, type of extract or fraction, Leishmania species used in the assays, parasite developmental stage evaluated, and IC_50_ value ranges. IC_50_ values were categorized into activity ranges following commonly used criteria in pharmacological and antiparasitic screening studies, where compounds with IC_50_ < 10 μg/mL are considered highly active, those between 10 and 50 μg/mL moderately active, 50–100 μg/mL weakly active, and >100 μg/mL inactive [97,115]. These thresholds provide a practical framework for comparing bioactivity across heterogeneous datasets, while IC_50_ remains a standard measure of compound potency in dose–response analyses [90]. Data shared across different published studies were integrated and visualized using a Circos-based framework implemented through the circlize package in the R version 4.5.3 (R Foundation for Statistical Computing, Vienna, Austria), as mentioned above.

This systematic review was prospectively registered on the Open Science Framework (OSF) platform, where the detailed protocol and registration information for the review, including search strategy, eligibility criteria, data extraction methods, and synthesis plan, can be accessed. The information can be accessed through the following link: https://osf.io/nxkt2/ accessed on 23 January 2026.

## 5. Conclusions

From the clinical practice and public health perspective, although the current evidence does not yet support direct therapeutic recommendations for humans, these findings provide a crucial foundation for developing alternatives to conventional leishmaniasis treatments. In tropical regions, plant biodiversity offers a strategic resource that should be integrated into public research agendas, governed by regulatory frameworks that ensure quality, reproducibility, safety, and environmental sustainability. Furthermore, research into plant-derived therapeutic agents may also promote local scientific capacity and foster sustainable, regional solutions for major neglected diseases, such as leishmaniasis and many others.

However, a substantial knowledge gap persists in the current literature, necessitating rigorous preclinical studies to facilitate progression toward clinical trials and translate findings into therapeutic and clinical applications. Currently, there is a trend in cellular and molecular pharmacology toward new strategies—such as nanoformulations, macrophage-targeted delivery systems, synergistic drug combinations, and in silico modeling—to identify safe and effective antiparasitic pharmacological candidates. Therefore, extracts and bioactive compounds from *A. indica* and *Cnidoscolus* spp. demonstrate potential for integration into modern pharmacological optimization platforms. However, the paucity of studies on *Cnidoscolus* spp. derivatives warrants further exploration.

Accordingly, future studies should evaluate a larger dataset of potentially effective species, investigate metabolic synergies within complex extracts, and systematically compare isolated compounds versus whole fractions. Ultimately, although this review provides a solid preclinical basis, the successful biotechnological development of natural product-based therapies will depend on rigorous, methodical, and clinically oriented future studies.

## Figures and Tables

**Table 1:** Individual characteristics of the studies included for analysis.

No.	Author/Year	Country	Design	Plant/Intervention	Part Used/Active Compound	Leishmania Species	Experimental Model	Outcomes Evaluated
1	Vijayakumar et al., 2025 [31]	India	In vitro/in silico	*A. indica.*	Leaves (azadiradione, pure compound)	*L. donovani*	Promastigotes, amastigotes, THP-1 monocyte cells	Moderate activity; Leishmania-specific drug potential of AZD.
2	López-Huerta et al., 2020 [22]	Mexico	In vitro/in vivo	*Cnidoscolus spinosus*	Leaves, hopane-type triterpenes (fraction CH_2_Cl_2_/MeOH)	*L. mexicana*	Promastigotes, Vero cells, CD-1 mice	Antileishmanial, anti-inflammatory activity
3	Juárez-Vázquez et al., 2020 [32]	Mexico	In vitro/in vivo	*Cnidoscolus* *tehuacanensis*	Lupeol acetate (Hexane extract)	*L. mexicana*	Promastigotes, amastigotes J774.2 macrophage cells, BALB/c mice	Leishmanicidal, anti-inflammatory activities
4	Fernandes et al., 2020 [33]	Brazil	In vitro	*Cnidoscolus quercifolius*	Leaves: linamarin (1), trans-cinnamic acid (2), β-sitosterol (3)/stigmasterol (4), and a mixture of 3-β-O-cinnamoyl-lupeol (5)/3-β-O-dihydrocinnamoyl-lupeol (6)	*L. braziliensis*	Promastigotes	Antileishmanial, cytotoxicity activity
5	Cesa et al., 2019 [34]	Italy	In vitro	*A. indica*	Commercial neem oil	*L. infantum*, *L. tropica*	Promastigotes, amastigotes, THP-1 cells	Antileishmanial, cytotoxicity activity
6	Mans et al., 2016 [35]	Suriname/Netherlands	In vitro	*A. indica*	Leaves (aqueous extract)	*L. guyanensis*, *L. donovani* *L. major*	Promastigotes, amastigotes, THP-1 monocyte cells	Antileishmanial activity, cytotoxicity
7	Dayakar et al., 2015 [36]	India	In vitro/in vivo	*A. indica*	Leaves (ethyl acetate fraction)	*L. donovani*	Promastigotes, amastigotes, THP-1 monocyte cells BALB/c mice	Antileishmanial activity, cytotoxicity, immunomodulation
8	Chouhan et al., 2015 [21]	India/Saudi Arabia	In vitro/in vivo	*A. indica*	Leaves and seeds (hexane fraction, ethanol, water)	*L. donovani*	Promastigotes, amastigotes, RAW 264.7 macrophage cells BALB/c mice	Antileishmanial activity, cytotoxicity, immunomodulation
9	Jumba et al., 2015 [37]	Kenya	In vitro/in vivo	*A. indica* *Ricinus* *communis*	Leaves (methanol fraction)	*L. major*	Promastigotes, amastigotes, peritoneal macrophage cells BALB/c mice	Antileishmanial activity, cytotoxicity
10	Kaur et al., 2013 [2]	India	In vivo	*A. indica* *Emblica* *officinalis*	Commercial product	*L. donovani*	BALB/c mice	Reduction in parasite load, immune response
11	García et al., 2012 [38]	Cuba	In vitro	*A. indica*	Leaves (hydroalcoholic extract)	*L. amazonensis*	Promastigotes and peritoneal macrophages from BALB/c mice	Antileishmanial
12	Carneiro et al., 2012 [20]	Brazil	In vitro	*A. indica*	Leaves and fruits (methanol-water extracts; hexane, dichloromethane, ethyl acetate fractions)	*L. amazonensis*	Promastigotes, amastigotes, Peritoneal macrophages, BALB/c mice	Antileishmanial activity, cytotoxicity
13	Singh et al., 2011 [39]	India	In vitro	*A. indica*	Leaves and bark (ethanolic extracts)	*L. donovani*	Promastigotes, Axenic amastigotes	Antileishmanial activity, Cytotoxicity
14	Khalid et al., 2005 [40]	Sudan	In vitro	*A. indica*	Leaves (methanolic extract)	*L. major*	Promastigotes	Antileishmanial

**Table 2:** Summary of plant derivatives, including compounds derived from *Azadirachta indica* and *Cnidoscolus* spp., evaluated against different *Leishmania* spp., both in vitro and in vivo.

Author/Year	Intervention	Leishmania Strain/Species (Homogeneous Code)	Model/State	Cells/Cell Line	In Vivo Animal Model	Antileishmanial Activity and Selectivity	Positive Control/Drug
Vijayakumar et al., 2025 [31]	Azadiradione (AZD) from *A. indica*	*L. donovani* (clinical isolate; code NR)	Promastigotes (in vitro)	THP-1	Not applicable	IC_50_ = 17.09 μM	Thioridazine (TRZ)/miltefosine
			Intracellular amastigotes			EC_50_ = 11.67 μM; CC_50_ = 56.32 μM; SI = 4.83	TRZ (positive)
López-Huerta et al., 2020 [22]	Hopanes 2–3 (*C. spinosus*)	*L. mexicana* (strain: “bricaire”; standard code NR)	Promastigotes (in vitro)	Not applicable	CD-1 (anti-inflammatory, not *Leishmania*)	Marginal activity; IC_50_ not explicitly reported	NR
Juárez-Vázquez et al., 2020 [32]	EH: Hexane extract LAF: Lupeol acetate-rich fraction (*C. tehuacanensis*)	*L. mexicana* (MNYC/BZ/62/M379) *L. mexicana*	Promastigotes (in vitro)	J774.2	Not applicable	EH: IC_50_ = 58.98 μg/mL; SI = 2.11; LAF: IC_50_ = 95.29 μg/mL	Miltefosine/ amphotericin B
			Amastigotes (in vitro)			EH: IC_50_ = 8.73 μg/mL; SI = 14.31 LAF: IC_50_ = 75.21 μg/mL	
	Lupeol acetate (LuAc) (*C. tehuacanensis*)		Promastigotes (in vitro)		BALB/c (anti-inflammatory, non-infectious)	Less active than extract; IC_50_ = 82.02 μg/mL	
			Amastigotes (in vitro)		Not applicable	IC_50_ = 52.33 μg/mL	Miltefosine/ amphotericin B
Fernandes et al., 2020 [33]	Linamarine (*C. quercifolius*)	*L. braziliensis* (MHOM/BR/2903)	Promastigotes (in vitro)	Not applicable	Not applicable	No inhibition at 31.25–2000 μg/mL; IC_50_ cannot be estimated	Amphotericin B
Cesa et al., 2019 [34]	Seed oil (*A. indica*)	*L. infantum* (MHOM/TN/80/IPT1)	Promastigotes (in vitro)	THP-1	Not applicable	IC_50_ = 215 μg/mL	Miltefosine
		*L. tropica* (MHOM/IT/2012/ISS3130)				IC_50_ = 211 μg/mL	
		*L. infantum* (MHOM/TN/80/IPT1)	Intracellular amastigotes			IC_50_ = 15.3 μg/mL; SI = 46	
		*L. tropica* (MHOM/IT/2012/ISS3130)				IC_50_ = 17.6 μg/mL; SI = 40	
Mans et al., 2016 [35]	Leaves, aqueous extract (*A. indica*)	*L. guyanensis* (AMC 2014)	Promastigotes (in vitro)	THP-1	Not applicable	IC_50_ > 500 μg/mL	Amphotericin B
		*L. major* (MHOM/IR/1972/NADIM5)				IC_50_ = 187 μg/mL	
		*L. donovani* (GEDII; Sudan)				IC_50_ = 247 μg/mL	
		*L. donovani* (BHU-814; India)	Intracellular amastigotes		Not applicable	IC_50_ = 451 μg/mL	
Dayakar et al., 2015 [36]	Leaves, ethyl acetate fraction (*A. indica*)	*L. donovani* (Dd8, ATCC)	Promastigotes (in vitro)	NR	Not applicable	IC_50_ = 52.4 μg/mL	Miltefosine
			Intracellular amastigote (In vitro)	THP-1 + PBMCs	Not applicable	parasite load ~45–50%; IC_50_ ≈ 10 μg/mL	Miltefosine (2.5 μM)
			Visceral leishmaniasis (In vivo)	Not applicable	BALB/c (5–6 weeks)	LDU spleen: 243.5 → 98.7; LDU liver: 4923.8 → 1354.6	Miltefosine (5 mg/kg)
Chouhan et al., 2015 [21]	ALE (ethanolic fractions of *A. indica* leaves)	*L. donovani* (MHOM/IN/83/AG83)	Promastigotes (in vitro)	Not applicable	Not applicable	IC_50_ = 34 μg/mL	Pentamidine
	ASE (ethanolic fraction of *A. indica* seeds)		Promastigotes (in vitro)	Not applicable		IC_50_ = 77.66 μg/mL	
	ALE (ethanolic fractions of *A. indica* leaves)		Intracellular amastigotes	RAW 264.7		IC_50_ = 17.66 μg/mL; CC_50_ = 461 μg/mL; SI = 26.10	
	ASE (ethanolic fraction of *A. indica* seeds)					IC_50_ = 24.66 μg/mL CC_50_ = 500 μg/mL	
	ALE/ASE (100–200 mg/kg)		Visceral leishmaniasis (In vivo)	Not applicable	BALB/c (6–8 weeks)	Protection (LDU): ALE: 87.76% liver/85.55% spleen; ASE: 85.54%/83.62%	Amphotericin B
Jumba et al., 2015 [37]	Methanolic extracts of *A. indica* leaves *and* *Ricinus communis* combined with Az + Rc	*L. major* (IDUB/KE/83 = NLB-144)	Promastigotes	Vero E6 cells (cytotoxicity) + BALB/c peritoneal macrophages (amastigotes)	Infected BALB/c mice (skin lesion on paw)	Promastigotes: IC_50_ *A. indica* = 10.1 µg/mL; combination = 12.2 µg/mL	Pentostam (IC_50_ 6.5 µg/mL) and amphotericin B (IC_50_ 4.5 µg/mL)
			Amastigotes	Vero E6 cells (cytotoxicity) + peritoneal BALB/c macrophages (amastigotes)	Not applicable	Amastigotes: IC_50_ *A. indica* = 11.5 µg/mL; combination = 9.0 µg/mL. CC_50_ cytotoxicity in Vero: *A. indica* = 149 µg/mL	
Kaur et al., 2013 [2]	*A. indica* (pure herb, 100–200 mg/kg oral)	*L. donovani* (MHOM/IN/80/Dd8)	Visceral leishmaniasis (In vivo)	Not applicable	BALB/c (5–6 weeks), infected intracardiacally	Significant reduction in hepatic parasite load measured in LDU; greater effect in combination with *E. officinalis*. IC_50_ is not reported	Sodium stibogluconate (SSG) 40 mg/kg
García et al., 2012 [38]	Hydroalcoholic extract (80% ethanol/20% water) of *A. indica* leaves	*L. amazonensis* (MHOM/77BR/LTB0016)	Promastigotes (in vitro; antiamastigote only for extracts with SI > 5)	BALB/c peritoneal macrophages (study cytotoxicity and antiamastigote activity)	Not applicable	IC_50_ promastigotes: >200 µg/mL (inactive) → was not evaluated against amastigotes because it did not meet selection criteria (SI > 5)	Pentamidine (positive control)
Carneiro et al., 2012 [20]	Leaves *A. indica:* Ethanolic fraction (EtOH-l); hexane fraction (Hex-l); dichloromethane fraction (CH_2_Cl_2_-l)	*L. amazonensis* (IFLA/BR/67/PH8)	Promastigotes 72 h after treatment	Not applicable	Not applicable	EtOH-l: IC_50_ = 38.0 µg/mL; SI = 1.53 Hex-l: IC_50_ = 8.5 µg/mL CH_2_Cl_2_-l: IC_50_ = 3.9 µg/mL; SI = 14.1	Amphotericin B
	*A. indica* Leaves: Ethanolic fraction (EtOH-l); dichloromethane fraction (CH_2_Cl_2_)		Intracellular amastigotes 72 h after treatment	Peritoneal BALB/c macrophages		EtOH-l: IC_50_ = 9.8 µg/mL; CC_50_ = 56.96 µg/mL; SI = 5.81 CH_2_Cl_2_-l: IC_50_ = 1.1 µg/mL; CC_50_ = 48.1 µg/mL; SI = 43.72	
	*A. indica* seeds: Chloroform fraction (CHCl_3_-s); Ethyl acetate fraction (EtOAc-s)		Promastigotes 72 h after treatment	Not applicable		CHCl_3_: IC_50_ = 1.2 µg/mL; SI = 8.3 EtOAc-s: IC_50_ = 1.4 µg/mL	
	*A. indica* seeds: Chloroform fraction (CHCl_3_-s)		Intracellular amastigotes 72 h after treatment	Peritoneal BALB/c macrophages		CHCl_3_-s: IC_50_ = 0.6 µg/mL; CC_50_ = 9.15 µg/mL; SI = 15.25	
	*A. indica* integument: Ethanolic fraction (EtOH-t); dichloromethane fraction (CH_2_Cl_2_-t)		Promastigotes 72 h after treatment	Not applicable		EtOH-t: IC_50_ = 2.7 µg/mL; SI = 12 CH_2_Cl_2_-t: IC_50_ = 2.1 µg/mL; SI = 15.9	
	*A. indica* integument: Ethanolic fraction (EtOH-t); dichloromethane fraction (CH_2_Cl_2_-t)		Intracellular amastigotes 72 h after treatment	Peritoneal BALB/c macrophages		EtOH-t: IC_50_ = 0.4 µg/mL; CC_50_ = 28.9; SI = 72.25 CH_2_Cl_2_-t: IC_50_ = 0.6 µg/mL; CC_50_ = 43.54; SI = 72.56	Amphotericin B
Singh et al., 2011 [39]	Crude soluble antigens (CSAs) from *A. indica* (leaves/bark/oil)	*L. donovani* (MHOM/IN/80/DD8)	Promastigotes (in vitro)	Not applicable	Not applicable	Minimum effective concentration (MEC) = 0.1 mg/mL (48 h). Does not report IC_50_ values	Amphotericin B (1 µg/mL); SAG (20 µg/mL)
	CSA from *A. indica*		Axenic amastigotes (in vitro)			Activity 1.9 times higher against amastigotes than promastigotes	Amphotericin B
Khalid et al., 2005 [40]	Crude methanolic extract (80%) of *Azadirachta indica* leaves	*L. major* (clinical isolate; code NR)	Promastigotes (in vitro)	Not applicable	Not applicable	LC_50_ = 10.21 µg/mL (considerable activity) Neither CC_50_ nor SI is reported	Pentostam (LC_50_ = 3.077 µg/mL)

NR = Not reported.

**Table 3:** Antileishmanial Activity of *Cnidoscolus* spp. Leaf Extracts and Fractions against Different Leishmania Species.

Extract or Fraction	Leishmania Species	Cell Line Used for Evaluation	Parasite Developmental Stage	IC_50_ Range (µg/mL)	Author/Year
Dichloromethane/Methanol	*L. mexicana*	Not applicable	Promastigotes	Not reported	(López-Huerta et al., 2020) [22]
Hexane extract	*L. mexicana*	Not applicable	Promastigotes	>50–100 (Low activity)	(Juárez-Vázquez et al., 2020) [32]
		Murine macrophages J774.2	Amastigotes	>1–10 (High activity)	
Lupeol acetate-rich fraction		Not applicable	Promastigotes	>50–100 (Low activity)	
		Murine macrophages J774.2	Amastigotes	>50–100 (Low activity)	
Purified lupeol acetate		Not applicable	Promastigotes	>50–100 (Low activity)	
		Murine macrophages J774.2	Amastigotes	>50–100 (Low activity)	
Ethanolic extract	*L. braziliensis*	Not applicable	Promastigotes	>10–50 (Moderate activity)	(Fernandes et al., 2021) [33]

**Table 4:** Antileishmanial Activity of *Azadirachta indica* Seed Oil Extracts and Fractions against Different Leishmania Species.

Extract or Fraction	Leishmania Species	Cell Line Used for Evaluation	Parasite Developmental Stage	IC_50_ Range (µg/mL)	Author/Year
Commercial seed oil	*L. infantum*	Human monocytic leukemia cell line (THP-1)	Amastigotes	>10–50 (Moderate activity)	(Cesa et al., 2019) [34]
		Not applicable	Promastigotes	>200 (Practically inactive)	
	*L. tropica*	Human monocytic leukemia cell line (THP-1)	Amastigotes	>10–50 (Moderate activity)	
		Not applicable	Promastigotes	>200 (Practically inactive)	
Ethanolic fraction	*L. donovani*	Murine macrophages RAW 264.7	Promastigotes	>50–100 (Low activity)	(Chouhan et al., 2015) [21]
		Murine macrophages RAW 264.7	Amastigotes	>10–50 (Moderate activity)	
Chloroform fraction	*L. amazonensis*	Not applicable	Promastigotes (24 h post-treatment)	>1–10 (High activity)	(Carneiro et al., 2012) [20]
		Not applicable	Promastigotes (48 h post treatment)	>1–10 (High activity)	
		Not applicable	Promastigotes (72 h post treatment)	>1–10 (High activity)	
		Peritoneal BALB/c macrophages	Amastigotes (72 h post treatment)	Very high activity: ≤1	
Ethyl acetate fraction	*L. amazonensis*	Not applicable	Promastigotes (24 h post treatment)	>1–10 (High activity)	
		Not applicable	Promastigotes (48 h post treatment)	>1–10 (High activity)	
		Not applicable	Promastigotes (72 h post treatment)	>1–10 (High activity)	

## Data Availability

The study did not report any data. CRediT Roles and Example Research Tasks That Could be Attributed to Them. Zenodo. https://doi.org/10.5281/zenodo.18421449.
